# Sonic Hedgehog Signaling in Cerebellar Development and Cancer

**DOI:** 10.3389/fcell.2022.864035

**Published:** 2022-04-29

**Authors:** Wanchen Wang, Ryo Shiraishi, Daisuke Kawauchi

**Affiliations:** ^1^ Department of Biochemistry and Cellular Biology, National Center of Neurology and Psychiatry (NCNP), Tokyo, Japan; ^2^ Department of NCNP Brain Physiology and Pathology, Graduate School of Medical and Dental Sciences, Tokyo Medical and Dental University, Tokyo, Japan

**Keywords:** sonic hedgehog, cerebellum, medulloblastoma, brain tumor, patched1, smoothened, GLI family

## Abstract

The sonic hedgehog (SHH) pathway regulates the development of the central nervous system in vertebrates. Aberrant regulation of SHH signaling pathways often causes neurodevelopmental diseases and brain tumors. In the cerebellum, SHH secreted by Purkinje cells is a potent mitogen for granule cell progenitors, which are the most abundant cell type in the mature brain. While a reduction in SHH signaling induces cerebellar structural abnormalities, such as hypoplasia in various genetic disorders, the constitutive activation of SHH signaling often induces medulloblastoma (MB), one of the most common pediatric malignant brain tumors. Based on the existing literature on canonical and non-canonical SHH signaling pathways, emerging basic and clinical studies are exploring novel therapeutic approaches for MB by targeting SHH signaling at distinct molecular levels. In this review, we discuss the present consensus on SHH signaling mechanisms, their roles in cerebellar development and tumorigenesis, and the recent advances in clinical trials for MB.

## 1 Introduction

In 1980, Nüsslein-Volhard and Wieschaus first identified the hedgehog (*hh*) gene in a *Drosophila* mutant by screening (Nusslein-Volhard and Wieschaus, 1980). *hh*-mutant larvae exhibited a disrupted body plan and duplicated denticles (cuticle projections in the anterior half of each body segment). The appearance of lawns of denticles sticking out of the segments resembled that of a hedgehog spine, inspiring the gene name. In 1992, the *Drosophila hh* gene was cloned by three independent groups (Lee et al., 1992; Mohler and Vani, 1992; Tabata et al., 1992). Shortly thereafter, homologs of the *hh* gene have been reported in vertebrates (Krauss et al., 1993; Riddle et al., 1993). In mammals, the HH family of proteins consists of three members: Sonic hedgehog (SHH), Desert hedgehog, and Indian hedgehog. Among them, SHH signaling has been the best studied so far and is known to play a central role in the regulation of developmental processes, such as the formation and function of the limb bud, nervous system, skeleton, and skin.

**FIGURE 1:**
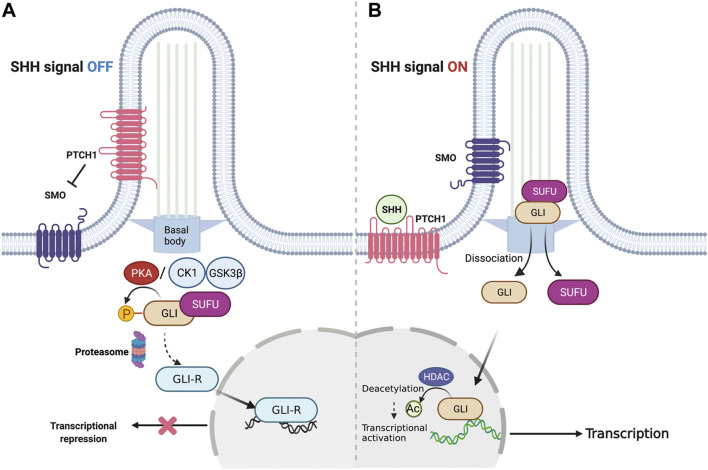
Overview of canonical SHH signaling. **(A)** Representative signaling cascade when SHH signaling is shut off. In the absence of the SHH ligand, PTCH1 gets localized to the plasma membrane in the primary cilium and inhibits SMO. This causes the GLI family proteins to stay restrained by SUFU to facilitate their phosphorylation by PKA. Consequently, the GLI proteins undergo proteolysis to generate GLI-R that suppresses the transcription of target genes. **(B)** Representative signaling cascade when the SHH signaling is switched on. In the presence of the SHH ligand, PTCH1 gets inhibited by the SHH ligand-dependent interaction. This activates SMO, allowing its translocation to the cilia. Subsequently, GLI proteins dissociate from SUFU to enter the cell nucleus and activate the target genes. SHH, Sonic hedgehog; PTCH1, Patched1; SMO, Smoothened; GLI, Glioma-associated oncogene; SUFU, Suppressor of fused; PKA, Protein kinase A.

The key components of canonical SHH signaling include the secreted ligand SHH, its twelve-pass transmembrane receptor patched1 (PTCH1), the class Frizzled seven-transmembrane helix G protein-coupled receptor, smoothened (SMO), and the downstream glioma-associated oncogene (GLI) family of transcription factors (Figure 1). After SHH binds to PTCH1, the primary cilium, a microtubule-based membrane protrusion functions as the central hub of signal initiation by releasing SMO from PTCH1-mediated signal repression. Immunolocalisation studies have revealed that the primary cilium harbors proteins required for signal transduction (Bangs and Anderson, 2017). Eventually, de-repressed SMO activates an intricate process mediated by the GLI family of zinc-finger transcription factors. In addition to these factors, several molecular mechanisms regulating these key components have been discovered at multiple levels of the non-canonical SHH pathway (Brennan et al., 2012), providing a deep understanding of how strictly and intricately SHH signaling is regulated in vertebrates.

The SHH signaling pathway in the vertebrate central nervous system has been studied well in mice (Chiang et al., 1996; Ruiz i Altaba et al., 2002). This pathway governs various neurodevelopmental processes within the brain. For example, SHH functions as a morphogen for cortical dorsal-ventral patterning and oligodendrogenesis in the early and later stages, respectively, and is required for the generation and maintenance of stem cell pools in postnatal and adult brains (Ahn and Joyner, 2005; Palma et al., 2005). The genetic ablation of ciliary genes induces abnormal radial astrocyte development and hypoplasia of the hippocampal dentate gyrus (Han et al., 2008). In the development of the spinal cord, SHH secreted from the notochord induces floor plate formation. Subsequently, SHH is secreted from the floor plate and functions as a long-range morphogen to trigger the differentiation of progenitor cells into spinal motor neurons and interneurons (Briscoe and Ericson, 2001; Danesin and Soula, 2017). SHH secreted from the floor plate also acts as a chemoattractant for axon pathfinding of commissural neurons (Charron et al., 2003). Thus, all these studies have confirmed the importance of SHH signaling in cell fate specification and axon pathfinding in the developing brain.

Another key role of SHH is to regulate cell proliferation in the dorsal brain as a potent mitogen (Dahmane et al., 2001). In particular, the cerebellum is one of the best-studied organs strongly affected by SHH signaling. Aberrant regulations of the signaling are related to neurodevelopmental diseases, including holoprosencephaly, Joubert syndrome (Aguilar et al., 2012), epilepsy (Hildebrand et al., 2016), autism spectrum disorder, and cancers, such as medulloblastoma (MB) (Northcott et al., 2012) and glioma (Clement et al., 2007). Thus, the importance of SHH signaling in the developing cerebellum has been increasingly recognized and research is focused on the pathway for the development of potential therapies for genetic diseases. In this article, we first review the roles of the principal components of SHH signaling pathways associated with normal cerebellar development and tumorigenesis at multiple levels of their regulatory mechanisms, and subsequently introduce potential therapeutic approaches with present clinical trials for SHH-driven cancer diseases based on recent accumulating knowledge.

## 2 Canonical Sonic Hedgehog Signaling Pathway

The *SHH* gene encodes a 45-kDa precursor protein. After translation, it is catalyzed by an autocleavage reaction, yielding an N-terminal fragment (SHH-N) that triggers the activation of its downstream cascade and a C-terminal fragment whose function is not yet known. The maturation of SHH-N is completed by the covalent coupling of cholesterol to its C-terminal end and subsequent palmitoylation at its N-terminus by Hedgehog acyltransferase, a membrane-bound acyltransferase (Tukachinsky et al., 2016; Qi et al., 2018). The mature SHH-N is anchored to the plasma membrane by cholesterol and released under the regulation of the Dispatched protein (Burke et al., 1999). It has also been reported that a metalloprotease, A disintegrin and metalloproteinase 17 contribute to SHH shedding from the cell membrane in the presence of a specific extracellular heparan sulfate (Dierker et al., 2009). Thus, SHH undergoes heavy modifications to exert its function at multiple levels (Dierker et al., 2009).

PTCH1 is an SHH receptor that negatively regulates canonical SHH signaling. Its expression is strictly regulated by a negative feedback loop of transcriptional regulation (Goodrich et al., 1996). Subsequently, an increasing level of PTCH1, together with another negative regulator, Hedgehog interacting protein 1 (HHIP1), facilitates to capture the SHH ligand for endocytosis and lysosomal degradation (Incardona et al., 2002), limiting the range of SHH action (Jeong and McMahon, 2005). In contrast, SHH has been reported to enhance the endocytosis of PTCH1 via SMURF1 and two HECT-domain ubiquitin E3 ligases (Yue et al., 2014), indicating the ligand-mediated post-transcriptional regulation of PTCH1. The ciliary localization of PTCH1 is critical for its function. PTCH1 is distributed in the primary cilia to prevent its downstream signaling activity without SHH stimulation. Once the SHH protein binds to PTCH1, it leaves the cilia and loses its inhibitory ability for SMO, initiating a canonical SHH signaling cascade in the primary cilia (Rohatgi et al., 2007). Thus, multiple regulatory mechanisms for PTCH1 determine the intensity of SHH signaling under normal conditions.

In addition to PTCH1, other SHH co-receptors, such as CDON, BOC, and GAS1 are also essential for SHH pathway activation (Beachy et al., 2010; Allen et al., 2011). Several reports have identified their functions in central nervous system (CNS) development, which include cell fate specification, axon guidance, and cell proliferation (Okada et al., 2006; Izzi et al., 2011; Kim et al., 2020). In neural tube development, they show overlapping functions, and triple knockout (KO) causes complete loss of SHH-dependent ventral spinal cord specification. Interestingly, only *Gas1* and *Boc*, not *Cdon* affect limb patterning, indicating a distinct requirement for co-factors in different tissues (Allen et al., 2011).

Signal transduction by SMO is a two-step process: ciliary localization and subsequent activation (Rohatgi et al., 2009). Similar to that of PTCH1, the ciliary localization of SMO is strictly regulated. SHH-mediated activation and constitutively active mutations of SMO promote its translocation to the cilia without changing its total expression level (Corbit et al., 2005). This translocation is mediated by the ArhGAP36-PKA-INVS axis (Zhang et al., 2019). In addition, the phosphorylation of the C-tail of SMO by casein kinase (CK) 1a and Grk2 under HH activation induces a conformational change, resulting in the ciliary accumulation of SMO (Zhao et al., 2007; Chen et al., 2010; Chen et al., 2011). As for the subsequent activation, given that the ciliary translocation of SMO is not sufficient for its activation (Rohatgi et al., 2009), it has long been a mystery as to how PTCH1 inhibits SMO without physical interactions. However, accumulating evidence has shown that a small fraction of membrane cholesterol, known as accessible cholesterol (Kinnebrew et al., 2019) could be a mediator between PTCH1 and SMO. Cholesterol acts as the endogenous ligand of SMO (Huang et al., 2016; Luchetti et al., 2016; Huang et al., 2018) via an ester bond on the Asp95 residue in its sequence (Xiao et al., 2017). Meanwhile, PTCH1 is capable of changing the distribution of cholesterol between the inner and outer leaflets of the plasma membrane (Zhang et al., 2018) and contributes to cholesterol cellular efflux as a cholesterol transporter (Bidet et al., 2011; Gong et al., 2018). Thus, multiple modification mechanisms influence the functions of SMO in SHH signaling.

The major target of activated SMO is the GLI family of zinc-finger transcription factors. In mammals, three members of the GLI family, GLI1, GLI2, and GLI3, mediate SHH-regulated transcription in an intricate manner (Ingham and McMahon, 2001). GLI1 acts as an amplifier and a downstream target of the SHH pathway (Marigo et al., 1996), whereas GLI2 and GLI3 exert dual effects on SHH activation depending on their post-transcriptional and post-translational modifications (Ruiz i Altaba et al., 2007). For example, in the absence of SHH signaling activation, a major negative regulator of the SHH pathway, suppressor of fused (SUFU) inhibits the GLI function by sequestering GLI in the cytoplasm (Humke et al., 2010). Subsequently, GLI2 and GLI3 undergo limited proteolysis by SCF^bTrCP^ ubiquitin E3 ligase and transform into their repressor forms (GLI2-R and GLI3-R) after phosphorylation is initiated by PKA (Humke et al., 2010; Tuson et al., 2011), CK1, and glycogen synthase kinase 3 (GSK3) (Pan et al., 2006; Tukachinsky et al., 2010). HH stimulation recruits the SUFU-GLI complex at the tip of the cilia, where the GLI dissociates from SUFU and translocates to the nucleus to activate the SHH pathway (Figure 1; Humke et al., 2010; Tukachinsky et al., 2010). In this process, the PKA phosphorylation sites of GLI2/3 are dephosphorylated; however, other sites are phosphorylated instead. GLI phosphorylation remodeling is crucial for adjusting the ratio of the GLI activator form (GLI-A) to repressor form (GLI-R) in the cell (Niewiadomski et al., 2014).

## 3 Non-Canonical Sonic Hedgehog Signaling Pathways

In addition to the classic canonical SHH pathway, the importance of non-canonical activation of the SHH pathway in development and tumorigenesis has been highlighted in emerging studies. Non-canonical SHH signaling has two distinct subtypes: 1) GLI-independent HH signaling, which can be further subdivided into SMO-independent and SMO-dependent and 2) alternative mechanisms inducing GLI activity (Robbins et al., 2012; Pietrobono et al., 2019). GLI- and SMO-independent mechanisms are often referred to as type I non-canonical HH signaling (Robbins et al., 2012). PTCH1 can be characterized as a dependence receptor because it is capable of inducing apoptotic cell death in the absence of SHH via another signaling pathway. While the PTCH1 C-terminal domain (CTD) is sufficient to induce cell death even in the presence of the SHH ligand (Mille et al., 2009), the insertion of a mutation that prevents caspase-mediated cleavage inhibited PTCH1-induced cell death (Thibert et al., 2003; Mille et al., 2009), demonstrating the functional significance of the CTD (Thibert et al., 2003). As SMO overexpression does not rescue cell death, this process is independent of the canonical SHH pathway (Thibert et al., 2003; Chinchilla et al., 2010).

In addition to the involvement in apoptotic cell death, PTCH1 negatively regulates cell proliferation by interacting with phosphorylated CCNB1, a G2/M checkpoint regulator, followed by shuttling it into the cytoplasm in an SMO-independent manner (Barnes et al., 2001). Supporting this study, the PTCH1 harboring a nonsense mutation found in basal cell carcinoma (BCC) failed to interact with CCNB1 (Barnes et al., 2005) and was capable of the morphological transformation of NIH3T3 fibroblast cells. Moreover, the CTD of PTCH1 phys(BCC) ically interacts with the ULK complex through ATG101 and impairs autophagic flux as a tumor suppressor (Chen et al., 2018). Taken together, loss of type I non-canonical HH signaling potentially contributes to tumorigenesis.

The type II non-canonical HH pathway is SMO-dependent. SMO has been shown to function not only as a classic SHH signaling transducer but also as a G protein-coupled receptor that catalyzes the GDP–GTP exchange of the G inhibitory (Gi) family of G proteins (Riobo et al., 2006). Several studies have indicated that SMO mediates the activation of small GTPases, such as Rac1 and RhoA, followed by the rearrangement of the actin cytoskeleton for the proper regulation of cell migration, angiogenesis, tubulogenesis, and synaptogenesis (Bijlsma et al., 2007; Renault et al., 2010; Sasaki et al., 2010; Polizio et al., 2011a; Polizio et al., 2011b). SRC-family kinases are also involved in SHH-mediated axon guidance without the activation of canonical SHH signaling (Yam et al., 2009).

Apart from the type I and type II non-canonical HH pathways, the aberrant activation of GLI bypasses the SHH–PTCH1–SMO route, which is also considered to be a part of the non-canonical HH pathway. These mechanisms, such as the RAS-RAF-MEK-ERK, PI3K-AKT-mTOR, and TGF-β pathways and epigenetic modulation (Pietrobono et al., 2019) allow the direct transcriptional activation of *GLI* and post-transcriptional modification of GLI. Our recent study further found that ependymoma-specific ZFTA fusion genes directly bind to the *GLI2* locus to activate it for tumor progression (Zheng et al., 2021). In addition, a study on SMO-resistant BCCs identified non-canonical GLI inducers, including AP-1, TGF-β, and their downstream Rho regulators (Yao et al., 2020). Thus, molecular analyses of cancerous cells provide novel insights into potential non-canonical SHH pathways.

## 4 Sonic Hedgehog Signaling in Cerebellar Development and Medulloblastoma Formation

The cerebellum is a prominent part of the hindbrain located in the dorsal brain stem and functions as the control center for motor coordination. It is known that the attenuation of SHH signaling by genetic mutations causes conditions of disrupted cerebellar development, such as hypoplasia, which is related to neurological diseases, including autism spectrum disorder (Filges et al., 2011), Joubert syndrome (De Mori et al., 2017), Dandy–Walker malformation (Zhang et al., 2021), and Down’s syndrome (Baxter et al., 2000; Roper et al., 2006). The aberrant activation of SHH signaling often induces extensive hyperplasia and results in MB, a common malignant pediatric brain tumor, arising in the cerebellum. Therefore, a profound understanding of how SHH signaling affects cerebellar development sheds light on novel therapeutic avenues for SHH-related genetic diseases.

**FIGURE 2:**
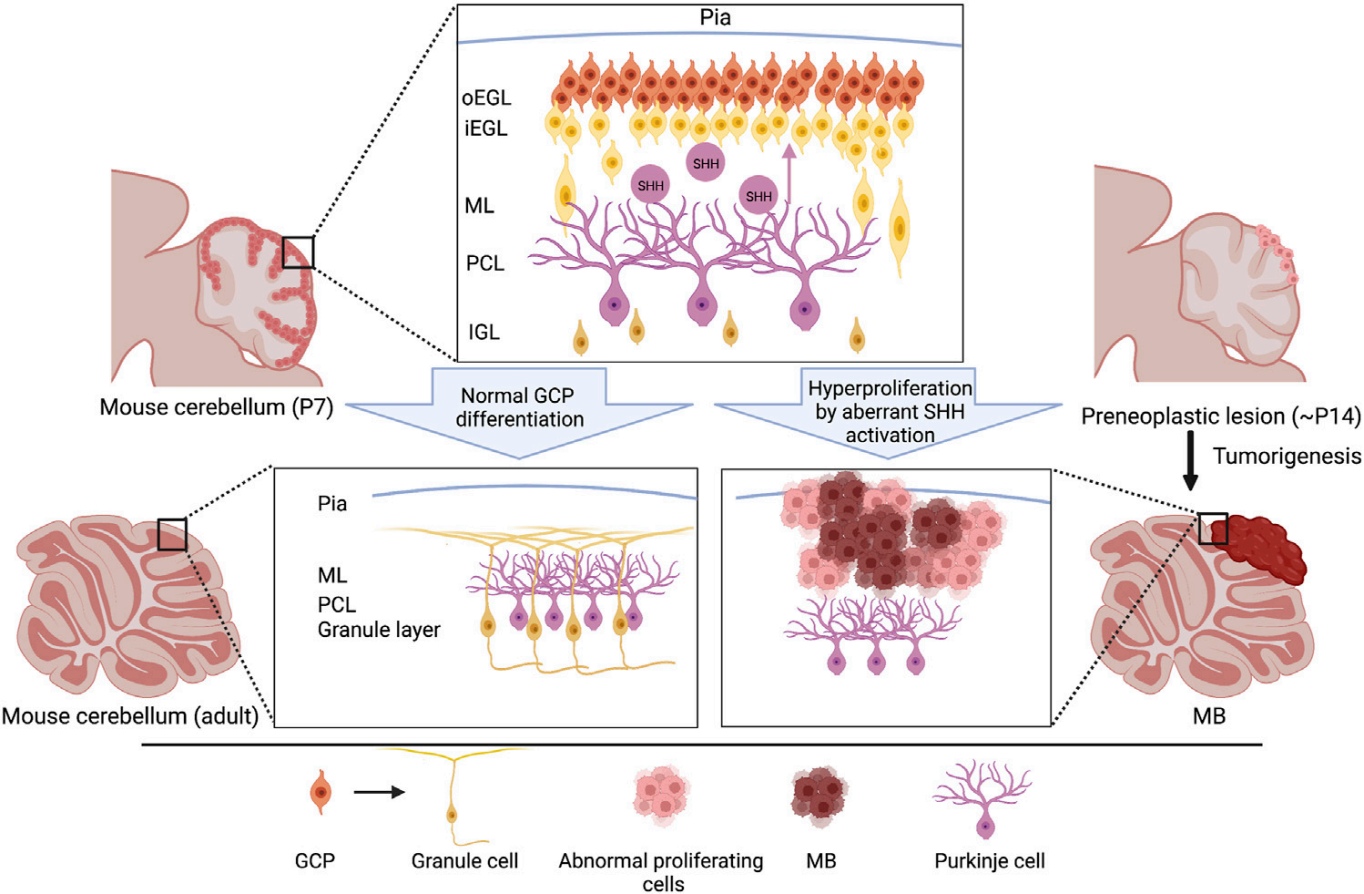
Schematic representation of cerebellar development and initiation of MB_SHH_. During the postnatal stage, GCPs normally proliferate in the outer edge of the EGL under the regulation of SHH secreted by the Purkinje cells. GCPs exit the cell cycle and subsequently migrate radially, toward the IGL, to mature into the iEGL. Somatic genetic mutations often cause aberrant SHH signaling that results in a failure to exit the cell cycle, leading to hyperproliferation. Abnormal differentiation of GCPs induces the formation of preneoplastic lesion and MB_SHH_s predominantly on the surface of the cerebellar hemispheres (bottom right). Please note that the locations of other subtypes of MBs, such as MB_WNT_s and MB_Grp3/4_s, are distinct from that of MB_SHH_s (Kawauchi et al., 2017), implying distinct cellular origins of these subgroups. GCP, granule cell precursor; oEGL, outer external granular layer; iEGL, inner external granular layer; ML, molecular layer; PCL, purkinje cell layer. IGL, internal granular layer.

Granule cells (GCs), interneurons essential for the coordination of afferent inputs and motor outputs of the cerebellum, represent the most prevalent neuronal type in the cerebellar system. During embryogenesis, GC progenitors (GCPs) arise from the upper rhombic lip, a germinal neuroepithelium in the most dorsal portion of the metencephalon, and migrate along the surface of the cerebellum to form the external granule layer (EGL) from E13 onward (Hatten and Heintz, 1995; Wang et al., 2005). After migration within the EGL at the postnatal stage, GCPs undergo a morphological transformation into round or polyhedral shapes and subsequent rapid proliferation, the strict regulation of which is critical for the development of the normal size and proper foliation of the cerebellum (Figure 2). SHH secreted from Purkinje cells (PCs) is the most efficacious mitogen for GCP expansion. Indeed, the local ablation of PCs inhibits GCP proliferation and induces thinning of the EGL (Smeyne et al., 1995). In contrast, the hyperactivation of SHH signaling due to a deficiency of *Ptch1* in GCPs caused abnormal proliferation and MB formation (Figure 2) (Schüller et al., 2008; Yang et al., 2008), indicating that SHH mediated an interplay between PCs and GCPs in the developing cerebellum. Consistent with the observation in *Ptch1*-deficient mice, the forced activation of SMO resulted in the hyperproliferation of GCPs, eventually resulting in high penetrance of MB formation (Hatton et al., 2008; Dey et al., 2012). Also, the genetic ablation of *Boc* and *Gas1* displayed their absolute contribution to the SHH-driven GCP proliferation by their direct interaction with PTCH1 (Izzi et al., 2011). In line with this report, elevated BOC levels enhanced SHH signaling activity in GCPs and mediated PTCH1-driven MB_SHH_ formation via CCND1-dependent DNA damage (Mille et al., 2014). Of note, a recent study revealed that SHH- but not IGF1-induced GCP proliferation causes replication stress via an increased replication fork speed and active fork density, a proxy of replication origin firing, potentially increasing the likelihood of MB-inducing mutations (Tamayo-Orrego et al., 2020). Thus, GCPs are susceptible to transformation into tumor cells by the hyperactivation of SHH signaling, while proliferating properly by the stimulation of PC-derived SHH ligands.

The roles of SUFU in GCP differentiation have been evaluated *in vivo* recently. While SUFU functions as either a sustainer or repressor of SHH signaling through the context-dependent regulation of GLI proteins, the conditional KO of *SUFU* explains its repressor function in GCPs. GCP-specific KO induces enhanced proliferation and delayed cell cycle exit by upregulating GLI2 and suppressing GLI3R, coinciding with *Fgf8* activation (Jiwani et al., 2020). However, unlike *Ptch1*, the loss of *Sufu* alone in GCPs is insufficient to cause MB development (Yin et al., 2019). This could be due to the destabilization of GLI2 in *Sufu*-KO GCPs. Indeed, the disruption of the Spop/Cul3-mediated proteasomal machinery for GLI2 remarkably enhanced MB formation at complete penetrance, indicating an essential role of GLI2 in tumorigenesis.

As a downstream molecule of GLI proteins, D-type cyclins have been identified as the proliferation machinery in GCPs (Kenney and Rowitch, 2000; Pogoriler et al., 2006; Tolosa et al., 2020). D-type cyclins interact with Cyclin-dependent kinase (CDK) 4 and phosphorylate retinoblastoma 1 (RB1) to inhibit its function, in turn allowing E2F-mediated gene expression for cell proliferation (Giacinti and Giordano, 2006). Indeed, *Rb1* conditional KO mice showed a delay in GCP differentiation (Marino et al., 2003; Shakhova et al., 2006), indicating the involvement of a D-type cyclin-RB1 axis in GCP proliferation *in vivo*. Interestingly, a recent study proposed that the CDK4-CCND1 complex also phosphorylates ATOH1, a basic helix-loop-helix (bHLH) transcriptional factor required for the commitment of GCP cell identity, at the Ser309 residue, which could enhance ATOH1 stability to maintain the undifferentiated state of GCPs (Miyashita et al., 2021). However, whether this model is completely independent of RB1 remains to be confirmed using Ser309 mutation-carrying primary GCPs.

*v-Myc myelocytomatosis viral related oncogene* (*MYCN*), another direct downstream target of GLI proteins (Kenney et al., 2003), also boosts GCP proliferation by negatively regulating cyclin-dependent inhibitors CDKN1B and CDKN2C (Knoepfler et al., 2002; Zindy et al., 2006). The previous studies have highlighted the role of MYCN in MB formation (Swartling et al., 2010; Kawauchi et al., 2012; Swartling et al., 2012). Furthermore, MYCN activates *miR-17–92* polycistronic miRNA clusters to maintain GCP proliferation (Northcott et al., 2009). Supporting this statement, mice lacking these miRNAs displayed decreased brain weight and abnormal foliation. In addition, *Ptch1*-driven MB formation was prevented by the conditional deletion of *miR-17–92* in GCPs, while a retroviral infection of either D-type cyclins, *Mycn*, or *miR-17–92* in *Ptch1* heterozygous GCPs enhanced MB formation (Uziel et al., 2009; Kawauchi et al., 2012; Zindy et al., 2014), indicating that these miRNAs are required for the initiation of MB formation. In addition, the silencing of *miR-17–92* and its orthologue *miR-106a–25* clusters by 8-mer LNA-modified antimiR oligonucleotides attenuated tumor growth of PTCH1-driven MBs (Murphy et al., 2013), indicating that these miR clusters are also important for both tumor initiation and progression.

Consistent with a previous finding that the primary cilium is involved in mammalian SHH signaling (see previous sections), the loss of cilium-related genes *Ift88* and *Kif3a* causes disrupted cerebellar development by the reduction of GCP expansion (Chizhikov et al., 2007; Spassky et al., 2008). A loss of ciliopathy genes, including *CEP290*, *Zfp423*, and *Talpid3*, has also been reported to attenuate SHH signaling activity, leading to cerebellar hypoplasia (Aguilar et al., 2012; Hong and Hamilton, 2016; Bashford and Subramanian, 2019). In contrast, Gαs, a G-coupled protein expressed at the primary cilium of the GCP, represses SHH signaling via the cAMP-dependent pathway and ciliary trafficking of GLI2 and SMO proteins (He et al., 2014). A recent study revealed that ATOH1 regulates ciliogenesis by upregulating *Cep131* and restoring centriolar satellite integrity, indicating an indirect contribution of ATOH1 to SHH signaling (Chang et al., 2019). It is also known that cilium-related genes are involved in MB formation. For example, the removal of the primary cilia by the loss of either *Ift88* or *Kif3a* inhibits either PTCH1- or SMO-driven MB formation and accelerates GLI2-induced tumorigenesis (Han et al., 2009; Wong et al., 2009; Han and Alvarez-Buylla, 2010; Barakat et al., 2013). Thus, ciliary formation affects tumorigenesis in an oncogenic driver-dependent manner. Furthermore, SHH-induced GCP proliferation has been reported to be enhanced by constitutive Gαi activation in the cilia (Barzi et al., 2011). Conversely, GCPs carrying deleted *Gnas* showed high proliferation and gave rise to MB_SHH_ (He et al., 2014). Thus, along with previous implications (Niewiadomski et al., 2013; Kool et al., 2014), G proteins are also involved in GCP proliferation and MB_SHH_ formation in the cilia.

Post-transcriptional regulation of SHH-related proteins has been reported to influence cerebellar development and tumorigenesis. Previous studies have shown that ERAP1 protects GLI from βTrCP-dependent degradation and activates the SHH pathway by binding to and sequestering USP47, a deubiquitylase enzyme of βTrCP. Consistent with this, the genetic or pharmacological inhibition of ERAP1 suppresses SHH-dependent tumor growth *in vitro* and *in vivo* (Bufalieri et al., 2019). In addition, the Itch/β-arrestin 2 complex has been reported to bind to SUFU and induce its K63-linked ubiquitylation. Without affecting the stability of SUFU, this process enhances the formation of the SUFU/GLI3 complex and consequently increases the amount of GLI3R, which keeps the SHH pathway switched off (Infante et al., 2018). Furthermore, histone deacetylase1 (HDAC1), a histone modifier, deacetylates GLI1 and GLI2 and enhances their transcriptional activation. Downstream of GLI proteins, MYCN is also regulated at the protein level by the E3 ubiquitin ligases HUWE1 and FBXW7 (Welcker et al., 2004; Zhao et al., 2008). While MYCN phosphorylated at Thr58 by GSK3b is recognized by FBXW7 for degradation (Yeh et al., 2004; Sjostrom et al., 2005), Aurora kinase A prevents FBXW7-mediated ubiquitination by directly interacting with MYCN. Intriguingly, the conditional inactivation of *Huwe1* and *Fbxw7* does not induce similar phenotypes in GCPs, which could imply differences in their other target proteins compared with MYCN (D'Arca et al., 2010; Jandke et al., 2011). In human MB_SHH_s, low *FBXW7* expression levels were correlated with poor prognosis (Suryo Rahmanto et al., 2016). Based on these studies, inhibitors that alter the catalytic action of SHH-regulating enzymes are presently being investigated as potential drugs for SHH-driven cancers, including MB.

Epigenetic regulation is thought to be important for strict SHH signaling tuning. For example, the bromo and extra C-terminal (BET) bromodomain protein BRD4, a transcriptional facilitator at super-enhancer sites (Whyte et al., 2013), is involved in GLI1 and GLI2 expression (Tang et al., 2014). shRNA-based *Brd4* knockdown and the pharmacological inhibition of BRD4 decreased GLI expression in SHH-driven MB cells (Tang et al., 2014). The histone modifier KDM6B has also been reported to regulate GCP proliferation via the epigenetic regulation of SHH-related genes. SHH signaling enhances the removal of a PRC2-mediated repressive H3K27me3 mark by KDM6B antagonizing PRC2 at the *Gli1* locus, resulting in the upregulation of *Gli1* and its downstream targets *Ccnd1* and *Mycn* in GCPs (Shi et al., 2014). In contrast, the *Kdm6b*-deficient cerebellum displayed a decreased cerebellar size and lack of foliation, which is reminiscent of the *Gli2*-KO cerebellum. The loss of *Kdm6b* further prevented SMO-driven MB formation, coincident with SHH downstream genes (Shi et al., 2014). Thus, KDM6B could be a key player in the epigenetic regulation of SHH signaling, although how the signaling pathway regulates KDM6B and other unidentified epigenetic factors in GCPs requires further investigation.

In addition to histone modifiers, chromatin remodelers have also been implicated as important in GCP proliferation. A loss of *Smarca4* (Zhan et al., 2011; Moreno et al., 2014; Shi et al., 2016), a critical component of the SWI/SNF chromatin remodeling complex, reduced EGL expansion and SMO-driven MB formation due to the lack of direct regulation of SHH-related genes. The conditional deletion of *Smarcb1*, another component of the SWI/SNF complex, did not upregulate SHH-, but WNT-related genes (Moreno et al., 2014) that impair GCP proliferation (Lorenz et al., 2011; Pei et al., 2012) and MB formation (Pöschl et al., 2014) by antagonizing SHH signaling. These results imply that these two SWI/SNF components may function differently in the GCPs.

Recent genome-wide data-mining studies on human MB_SHH_s have identified novel machinery that regulates SHH signaling. In addition to GNAS in the primary cilia (see previous sections), mutations in non-coding U1 small nuclear RNAs have been identified as potential regulators of SHH signaling in human MB_SHH_s (Suzuki et al., 2019). These mutations are strongly associated with the aberrant splicing of SHH-related genes, which in turn activates them abnormally. Thus, multi-disciplinary studies on human diseases would provide further insights into the unexpected regulatory mechanisms of SHH signaling pathways in the future.

Other types of cerebellar cells exhibit SHH signaling activity in *Ptch1*-, *Gli1*-, and *Gli2*-reporter mice (Bai et al., 2002; Corrales et al., 2004; Lewis et al., 2004). In addition to GCPs/GCs, TNC-positive GABAergic intermediate progenitors, astrocyte precursors in the white matter, and Bergmann glia exhibit SHH activity at postnatal stages. Indeed, SHH from PCs stimulates the proliferation of these white matter progenitors, and the conditional deletion of *SMO* in these progenitors reduces their proliferation and expansion (Fleming et al., 2013). Unlike that in white matter progenitors, the deletion of *SMO* in the Bergmann glia indirectly affects GCP proliferation with abnormal cytoarchitecture but not their own proliferative defects (Cheng et al., 2018). Whether these cell types are the cellular origin of MB_SHH_ remains to be explored.

## 5 Sonic Hedgehog Signaling Perturbation as Medulloblastoma Treatment

### 5.1 Prevalence of Genetic Predisposition in Human MB_SHH_s

The dysregulation of potent mitogens often results in cancer. As mentioned previously, the aberrant activation of SHH signaling is strongly associated with the development of MBs, which account for approximately 25% of all brain tumors in children. Thus far, in addition to MB_SHH_s, MBs consist of the other three subgroups (WNT, group 3, and group 4) according to their molecular characteristics. MB_SHH_s account for 30% of all MBs (Maier et al., 2021). In patients with MB_SHH_s, mutations in genes involved in these pathways cause the constant activation of the SHH pathway (*SHH* gene amplification, 3%; *PTCH1* loss-of-function mutation, 49.6%; *SMO-*activating mutation, 17.3%; *SUFU* loss-of-function mutation, 14.3%; *GLI2* gene amplification, 7.5%; and *MYCN* gene amplification, 12.8%) (Kool et al., 2014). Interestingly, the frequency of these mutations depends on the age of the patient: *PTCH1* mutations are found in all age groups, whereas *SUFU* mutations are mainly found in infants (0–3 years), and *SMO* mutations are predominantly detected in adults (18 years or older). Genetic amplification of *GLI2* and *MYCN* is mainly observed in children (4–17 years) and is often accompanied by *TP53* mutations, which are included in the high-risk group (Kool et al., 2014). Recent international multicenter MB predisposition studies have confirmed germline mutations in *SUFU*, *PTCH1*, and *TP53* and discovered novel predisposition genes, such as *ELP1* (14.4%), *BRCA2* (2.5%), and *PALB2* (1.0%), with the highest prevalence in MB_SHH_s patients (Waszak et al., 2018; Waszak et al., 2020). Whether these novel mutations affect SHH signaling and MB formation remains to be elucidated.

### 5.2 Smoothened Inhibitors

**FIGURE 3:**
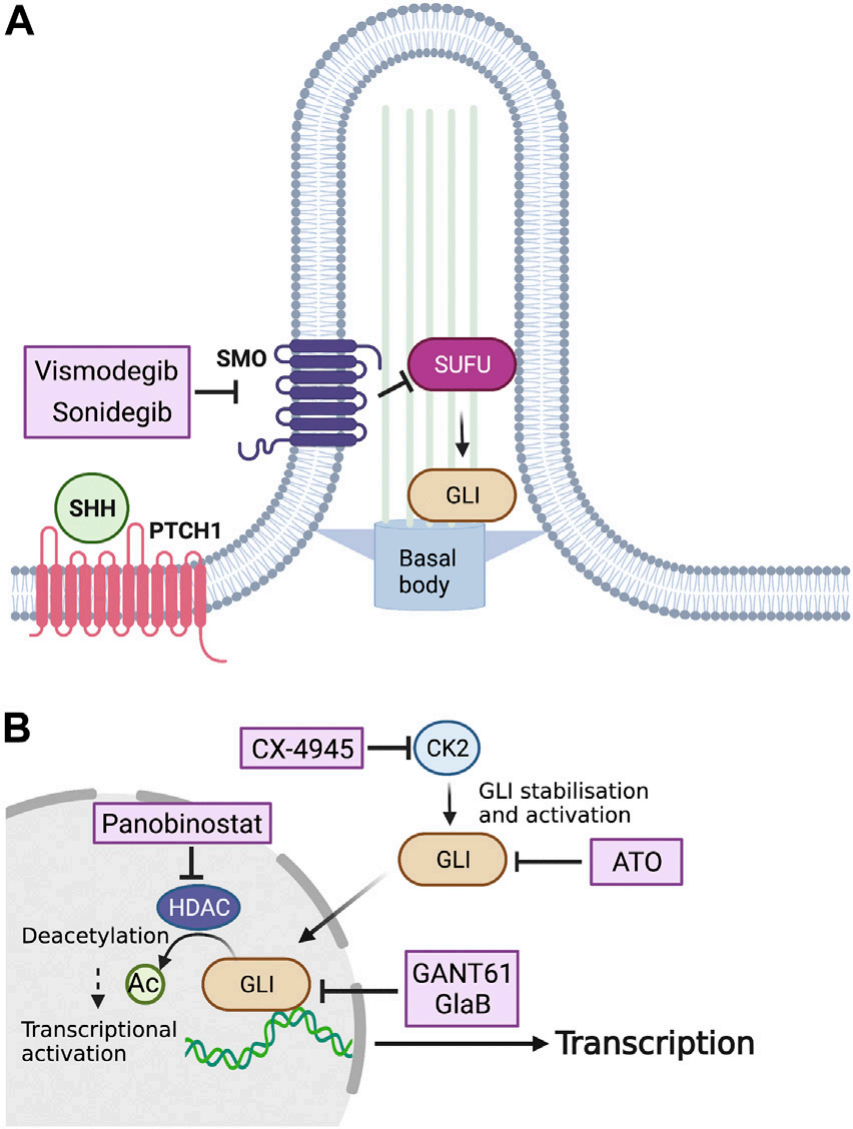
Potential inhibitors of SHH signaling for the treatment of MBs. **(A)** Vismodegib and sonidegib, drugs that inhibit SHH activation by SMO on the surface of the tumor cell, have been approved for treating MB patients. **(B)** Inside the tumor cell, silmitasertib (CX-4945) blocks the kinase activity of CK2, which results in dephosphorylation of GLI proteins, while GANT61, ATO, and GlaB are capable of preventing GLI-mediated transcription. Panobinostat, an HDAC inhibitor, reduces GLI activity via regulation of its acetylation. CK2, Casein kinase 2; GANT61, GLI antagonist 61; ATO, Arsenic trioxide; GlaB, Glabrescione B.

So far, several small molecules have been considered as a candidate drugs for SHH-driven tumors (Figure 3). Based on the high frequency of *PTCH1* mutations in MB_SHH_s, SMO inhibitors are promising therapeutic drugs against SHH-mediated cancers. Vismodegib (GDC-0449) is an SMO inhibitor approved by the U.S. Food and Drug Administration (FDA) as a first-line drug for the treatment of BCCs, which are also driven by hyperactivation of the SHH pathway. Vismodegib binds to the transmembrane domain of SMO and inhibits its downstream signaling, and has been shown to regress tumors in an allogeneic transplantation model of tumor cells in the MB_SHH_ mouse model (Robarge et al., 2009). Clinical trials of four drugs with potential clinical applications are presently underway (NCT01878617, NCT00939484, NCT01239316, and NCT00822458). In addition to vismodegib, sonidegib (LDE-225) has also been used in clinical trials as an SMO inhibitor that binds to the transmembrane domain (NCT01708174, NCT01208831, NCT00880308, NCT01125800, and NCT03434262). Sonidegib is more potent than vismodegib in inhibiting SMO and has been shown to cross the blood-brain barrier, which has attracted much attention (Pan et al., 2010). Given the present development of various SMO inhibitors in addition to vismodegib and sonidegib (Lospinoso Severini et al., 2020), it is likely that new SMO inhibitors will continue to enter clinical trials. The therapeutic effects of SMO inhibitors are often only transient, however. The SMO D473H mutation has been found in recurrent tumors after continuous treatment with vismodegib and has been shown to confer drug resistance to vismodegib (Yauch et al., 2009). In addition, mutations in genes downstream of SMO, such as *SUFU*, *GLI*, and *MYCN*, can confer resistance to SMO inhibitors in MB_SHH_s (Kool et al., 2014). Furthermore, the activation of the phosphatidylinositol-3 kinase pathway has been shown to attenuate the effect of sonidegib in mice models (Buonamici et al., 2010). Thus, although SMO inhibitors are expected to be therapeutic agents for MB_SHH_s, congenital or acquired drug resistance remains a major issue. To overcome this difficulty, it is necessary to further examine the mechanism of resistance acquisition to develop molecular-targeted compounds for combinatorial drug therapy.

### 5.3 GLI Inhibitors

Because SMO is one of the most upstream targets of the SHH pathway, its inhibitors are not expected to have therapeutic effects on tumors with downstream genetic mutations. Therefore, in addition to SMO inhibitors, direct and indirect inhibitors of GLI proteins are presently under development. The first example of an indirect GLI inhibition is the inhibition of HDACs. As mentioned previously, GLI1 and GLI2 are known to increase their transcriptional activities via deacetylation by HDACs (Canettieri et al., 2010). A proof-of-concept validation using the MB_SHH_ mouse model showed that the oral administration of mocetinostat, an HDAC inhibitor, drastically suppressed tumor growth (Coni et al., 2017). In addition, the HDAC inhibitor panobinostat (MTX110), which has been approved by the FDA for the treatment of cancer, has recently entered clinical trials for recurrent MBs (NCT04315064), and is expected to be approved for clinical applications.

The second method of indirect GLI inhibition is the blockade of CK2 that stabilizes the GLI2 protein and increases its transcriptional activity (Purzner et al., 2018). *In vitro*, CX-4945, a highly specific inhibitor of CK2, was shown to inhibit CK2 in mouse MB_SHH_ cells and patient-derived tumor cells. *In vivo*, CX-4945 exhibits antitumor activity in orthotopic transplantation models of cancer cells derived from a vismodegib-resistant MB_SHH_ mouse model (Purzner et al., 2018). CX-4945 is presently undergoing clinical trials (NCT03904862).

Although no direct inhibitors of GLI proteins have undergone clinical trials to date, some have already been utilized in cancer research. GLI antagonist 61 (GANT61) is a small molecule that binds to the groove between the zing-finger domain of GLI and directly inhibits its transcriptional activity (Agyeman et al., 2014). It has been reported that GANT61 inhibits migration, invasion, and proliferation of human MB_SHH_ cell lines and promotes apoptosis (Lin et al., 2016). Glabrescione B (GlaB), an isoflavone naturally found in Derris glabrescens, also binds to the zinc-finger domain of GLI1 and interferes with its DNA-binding capability (Infante et al., 2015). It is also proven that GlaB inhibited MB_SHH_ growth *in vivo*, when delivered by polymer micelles and intraperitoneal injections (Infante et al., 2021). Arsenic trioxide (ATO) is another drug that directly binds to and inhibits GLI1 (Beauchamp et al., 2011) and GLI2 (Kim et al., 2010) and is approved by the FDA for the treatment of patients with acute promyelocytic leukemia (Raffoux et al., 2003). ATO inhibits the SHH pathway in human MB_SHH_ cell lines and prolongs the survival of a mouse model of MB_SHH_ (Beauchamp et al., 2011). It is hoped that further preclinical studies using patient-derived tissue xenografts will allow these drugs to enter clinical trials as novel therapeutic agents against MB_SHH_.

## 5.4 CDK Inhibitors

Finally, we will discuss the therapeutic potential of CDK inhibitors in MB_SHH_. Palbociclib, an inhibitor of CDK4 and CDK6, is approved by the FDA for the treatment of patients with advanced breast cancer (Turner et al., 2018). The CDK4/6-Cyclin D-RB1 pathway is important for cell-cycle progression (Goel et al., 2018) and is expected to be a therapeutic agent for various types of cancers. As a downstream target of SHH signaling, the expression of *CDK6* is elevated in human MBs and its pharmacological inhibition by palbociclib resulted in attenuated tumor growth and prolonged survival in an MB_SHH_ mouse model (Raleigh et al., 2018). Palbociclib is presently in clinical trials for the treatment of several types of pediatric brain tumors, including MB (NCT 02255461). A recent phase I trial of palbociclib in pediatric brain tumor patients defined a dosage of 75 mg/m^2^ for 21 consecutive days as an optimal condition (Van Mater et al., 2021). In addition to palbociclib, a recent study has revealed that THZ-1, a covalent inhibitor of CDK7, antagonizes GLI1 and GLI2 transcription, preventing the growth of SHH-driven MBs and SMO inhibitor-resistant human tumor cell lines *in vivo* and *in vitro* (Liu et al., 2019). Thus, CDK inhibitor-based inhibition of the SHH pathway would be an alternative therapeutic strategy for SMO inhibitor-resistant tumors.

## 6 Discussion

To date, tremendous efforts have been made to reveal the molecular mechanisms involved in the SHH pathway. Meanwhile, exciting new insights into the molecular mechanism underlying the SHH pathway are constantly being updated. For example, it has been reported that the SHH ligand is delivered by a tiny cellular projection called cytoneme, one of the most novel cellular compartments, using sophisticated live imaging technology (Hall et al., 2021). Despite such emerging new findings, we have encountered many obstacles in treating MB_SHH_s in the clinical field. The inter- and intra-tumor heterogeneity with different SHH-related mutations suggests the need for personalized treatment (Kool et al., 2014). MB_SHH_-specific mutations could interplay with SHH signaling to accelerate tumor progression. Actually, our recent study has reported that BCOR, a main component of PRC1.1 is frequently mutated in human MB_SHH_s; its deletion increases *Igf2* transcriptional activation via histone H2A modification, causing enhanced tumor progression in SHH-driven murine tumors (Kutscher et al., 2020). Moreover, a recent single-cell genomics study identified *Hes1* and *Myod* as markers for MB_SHH_ cells responsive and resistant to an SHH inhibitor (Ocasio et al., 2019), respectively, implying the existence of unknown crosstalk between SHH signaling and these genes. Thus, recent advanced experimental techniques and multidisciplinary researches would offer the opportunities to understand the SHH signaling network more deeply, providing not only better biological insights into the normal development of organisms, but also opportunities to develop novel therapeutic avenues for many SHH-driven cancers.

## Abbreviations

HH, Hedgehog; SHH, Sonic hedgehog, PTCH1, Patched1; SMO, Smoothened; GLI, Glioma-associated oncogene; SUFU, Suppressor of fused; HHIP1, Hedgehog Interacting Protein 1; PKA, Protein kinase A; MYCN, v-myc myelocytomatosis viral related oncogene; KO, Knockout; MB, Medulloblastoma, GCP, Granule cell progenitor, EGL, External granule layer; RB1, Retinoblastoma 1; FDA, Food and Drug Administration; CDK, Cyclin-dependent kinase; CK, Casein kinase; GANT61, GLI antagonist 61; ATO, Arsenic trioxide; GlaB, Glabrescione B; BCC, Basal cell carcinoma; PC, Purkinje cell.
